# Damage-free LED lithography for atomically thin 2D material devices

**DOI:** 10.1038/s41598-023-29281-w

**Published:** 2023-02-14

**Authors:** Yue Shi, Takaaki Taniguchi, Ki-Nam Byun, Daiki Kurimoto, Eisuke Yamamoto, Makoto Kobayashi, Kazuhito Tsukagoshi, Minoru Osada

**Affiliations:** 1 Department of Materials Chemistry and Institute of Materials and Systems for Sustainability (IMaSS), Nagoya University, Nagoya, 464-8601 Japan; 2 International Center for Materials Nanoarchitectonics (WPI-MANA), National Institute for Materials Science (NIMS), Tsukuba, 305-0044 Japan

**Keywords:** Nanoscale devices, Electronic devices, Techniques and instrumentation, Surface patterning

## Abstract

Desired electrode patterning on two-dimensional (2D) materials is a foremost step for realizing the full potentials of 2D materials in electronic devices. Here, we introduce an approach for damage-free, on-demand manufacturing of 2D material devices using light-emitting diode (LED) lithography. The advantage of this method lies in mild photolithography by simply combining an ordinary optical microscope with a commercially available LED projector; the low-energy red component is utilized for optical characterization and alignment of devices, whereas the high-energy blue component is utilized for photoresist exposure and development of personal computer designed electrode patterns. This method offers maskless, damage-free photolithography, which is particularly suitable for 2D materials that are sensitive to conventional lithography. We applied this LED lithography to device fabrication of selected nanosheets (MoS_2_, graphene oxides and RuO_2_), and achieved damage-free lithography of various patterned electrodes with feature sizes as small as 1–2 μm. The LED lithography offers a useful approach for cost-effective mild lithography without any costly instruments, high vacuum, or complex operation.

## Introduction

Recent advances in 2D materials provide great promise for next-generation electronics owing to their unique properties at atomic thickness^1–6^. Desired electrode patterning on 2D materials is an essential and foremost step for realizing new electronic devices. Photolithography and electron beam (EB) lithography are well-established techniques for site-specific and on-demand nanofabrication with high-resolution patterning. However, these techniques have some drawbacks, such as high cost of instruments, high vacuum, complex operation, low throughput, and processing damage, which significantly limit their utility. For example, conventional photolithography (or UV lithography) normally requires the premanufacture of custom-made hard photomasks, which are very expensive, time consuming to produce and often limit the flexibility. The EB lithography often causes inadvertent impurity doping and crystal damage^7–9^; the atomically thin nature of 2D materials renders them prone to beam damage during the EB lithography and to degradation of their superior electrical properties. Without addressing these issues, the potential advantages of employing 2D materials may be greatly reduced. The development of facile mild lithography that enables damage-free, on-demand manufacturing of 2D material devices without costly instruments or complex operation is urgently needed.

Here, we present a strategy for damage-free, on-demand manufacturing of 2D material devices using light-emitting diode (LED) lithography. Our LED lithography system is quite simple, simply combining an ordinary optical microscope with a commercially available LED projector; the low-energy red component is utilized for optical characterization and alignment of devices, whereas the high-energy blue component is utilized for photoresist exposure and development of PC-designed electrode patterns. This method enables maskless mild lithography; the designated pattern can precisely be located and rapidly developed on the photoresist layers so that maskless photolithography can be achieved, free from the process damage often encountered in the conventional lithography. We applied this LED lithography to device fabrication of selected nanosheets (MoS_2_, RuO_2_ and graphene oxides) as model systems, and successfully achieved damage-free lithography of various patterned electrodes with feature sizes as small as 1–2 μm.

## Results and discussion

### Device fabrication based on LED lithography

**Figure 1:**
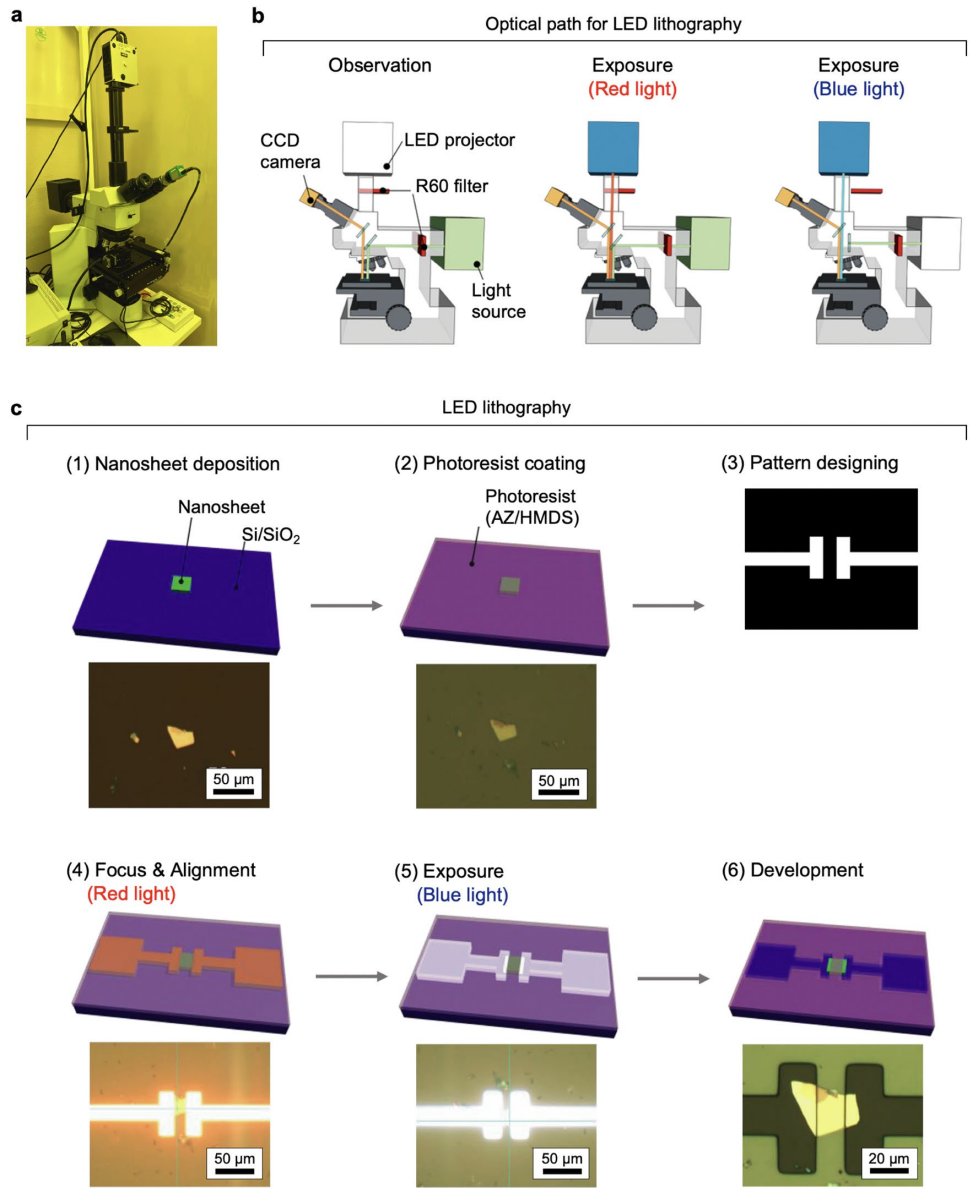
LED lithography. (**a**) Photograph of the LED lithography. (**b**) Schematic illustration of the optical pass for the LED lithography. (**c**) A typical procedure for the LED lithography. The upper part shows a schematic illustration for six steps, together with the corresponding photographs (lower).

Figure 1a shows a photograph of the LED lithography system. The LED lithography system is mainly composed of four parts: an optical microscope, an LED projector, a computer, and a charge-coupled device (CCD) camera, where the camera and projector parts are connected to ocular and adapter lenses, respectively. To protect the photoresist layer from unexpected exposure, two red sharp cut filters (R60, ≤ 600 nm) were applied to the light sources of the optical microscope and the LED projector. The details of the optical paths are illustrated in Fig. 1b. During observation, the light from the projector was cut off, and the optical microscope could provide observation of the nanosheet position for address-free pattern alignment of the nanosheets. During exposure, the light from the microscope was cut off so that the patterns at the designated location could be exposed by the LED projector.

**Figure 2:**
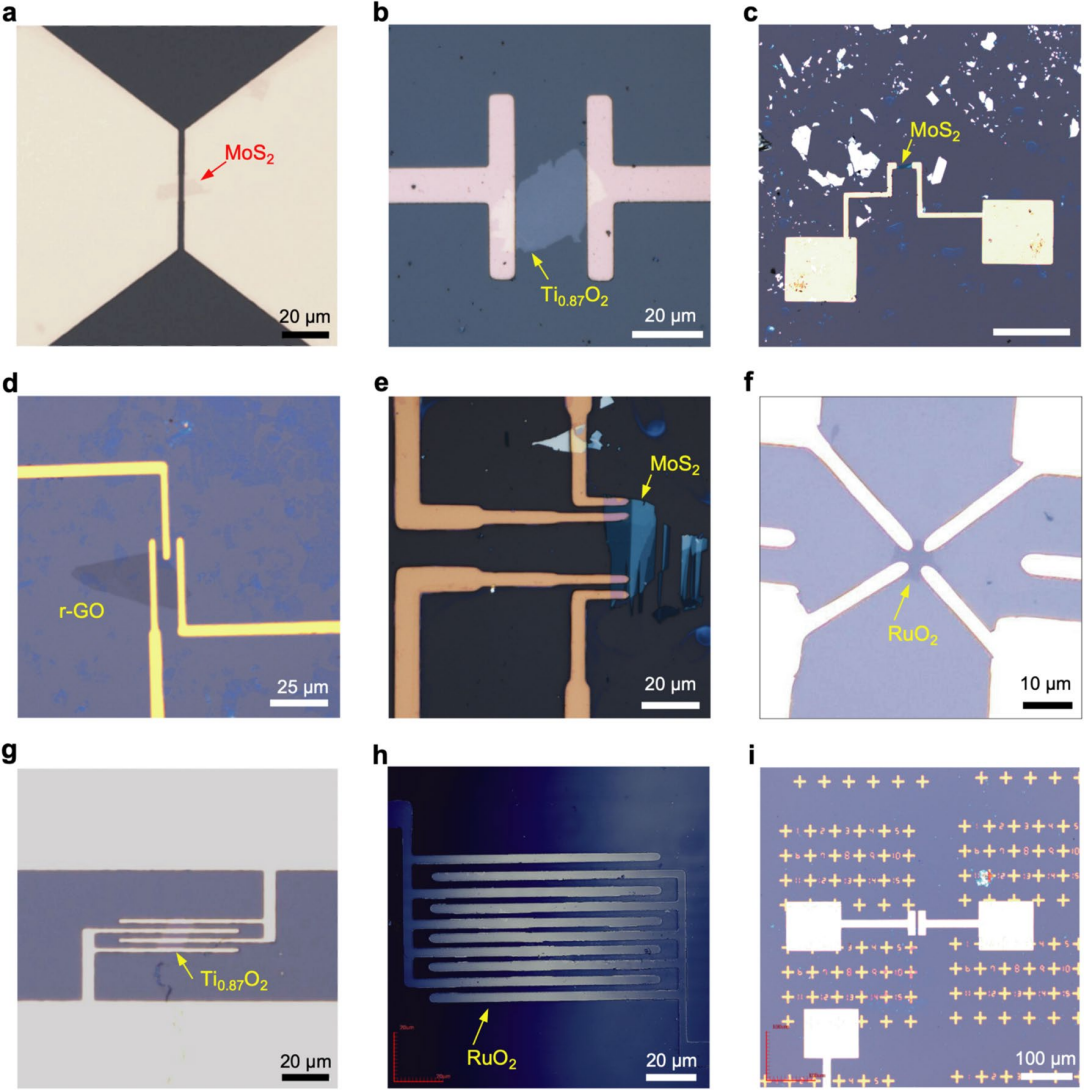
2D nanosheet devices fabricated by the LED lithography. (**a**) Two-terminal device of 1L MoS_2_. (**b**) Two-terminal device of 1 L Ti_0.87_O_2_. (**c**) Two-terminal device of 1 L MoS_2_. (**d**) Three-terminal device of 1 L r-GO. (**e**) Four-terminal device of 1 L MoS_2_. (**f**) van der Pauw device of 1 L RuO_2_. (**g**) Four-terminal device of 1 L Ti_0.87_O_2_. (**h**) Interdigitated device of 1L RuO_2_ film. (**i**) Address patterns.

We applied the LED lithography to pattern the metal contacts of nanosheet devices (Fig. 1c). The process for LED lithography includes six steps: (1) nanosheet deposition, (2) photoresist coating, (3) pattern design, (4) alignment using red light, (5) exposure to blue light and (6) development. The process began by depositing 2D nanosheets on 90- or 290-nm SiO_2_/Si substrates via drop casting. Then, hexamethyldisilazane (HMDS) was spin-coated on the sample surface as a hydrophobic treatment to enhance the adhesion of the subsequent photoresist layer. We chose AZ1500 (4.4 cp, Merck group, USA) as a photoresist^10^ because of its blue-light sensitivity and good compatibility with the developer as it can be easily developed without residues. A 500-nm-thick photoresist layer was deposited by spin-coating it on the HMDS layer and baking the film at 90 °C for 3 min. After film preparation, the pattern of metal contacts designed by a PC was aligned by optical microscope, and the position and focusing of the pattern were checked under exposure to red light. After fine adjustment, the high-energy blue component was employed for the exposure of the photoresist layer. Finally, the exposed region of the photoresist layer was removed to form the mask for the patterned metal contacts, followed the development process with tetramethylammonium hydroxide (NMD-3) solution (2.38%). After the LED lithography, a metal layer (such as Au or Ti/Au) was deposited by electron beam evaporation on the developed film. The resultant film was then immersed in acetone at 50 °C for 1 h to lift off the extra metal coating. Figure 2 shows several examples of 2D devices fabricated by the LED lithography. Through PC-based mask design and appropriate alignment, we successfully fabricated several types of 2D devices, including two-terminal, multiterminal, and interdigitated devices, and designed patterns (Supplementary Fig. 1).

### Characterization of patterned microstructures

**Figure 3:**
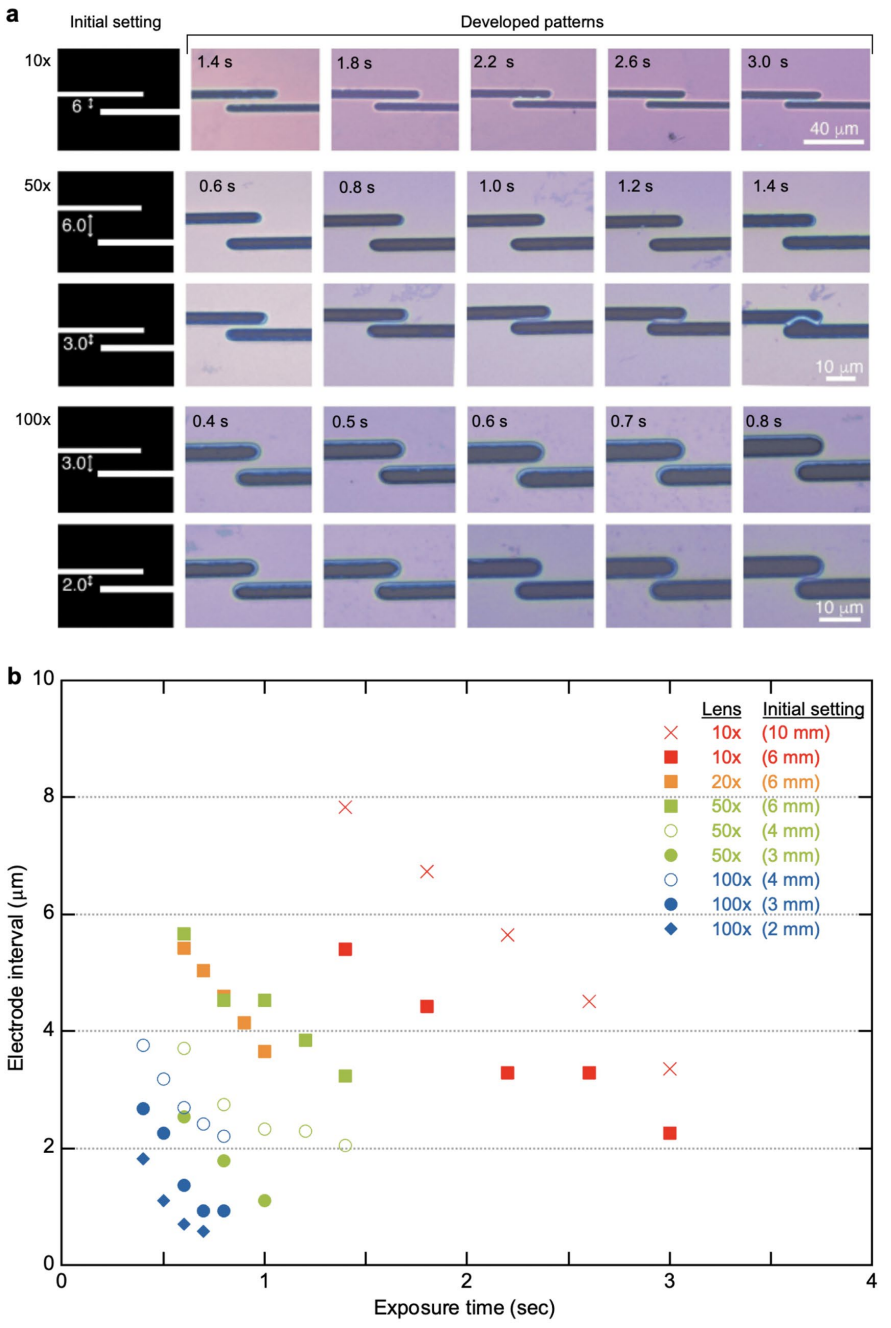
Patterning capability. (**a**) The dependence of the developed patterns on the exposure condition in comparison with the initially designed patterns (left). Two electrodes fabricated by LED lithography with different objective lenses (×10, ×50 and ×100) and various exposure time. (**b**) The relationship between the electrode interval and the exposure time. We evaluated the electrode intervals fabricated by different objective lenses with various initial settings.

To demonstrate the patterning capability, we investigated the exposure conditions using four different objective lenses (with 10×, 20×, 50× and 100× magnifications). Figure 3a highlights the dependence of the developed patterns on the exposure condition in comparison with the initially designed patterns (left). The patterning capability and device structures were characterized by the confocal laser microscopy. All data are shown in Supplementary Figs. 2–6. Clearly, the magnification of the lens strongly affects both the development rate and resolution. Since the power density of the exposure light is inversely proportional to the spot diameter, higher magnification lenses yield enhanced power densities, resulting in faster development. A short exposure time was needed for higher magnifications. We also note that exposures with different magnifications can produce electrodes with various feature sizes and gaps. Figure 3b illustrates the influence of the exposure time on the electrode interval. For a pair of electrode patterns with a set distance, a prolonged exposure time would result in shrinkage of the electrode interval. For the lower magnifications (10× and 20×), the minimum gap size reached 3 μm. The use of higher magnifications (50 × and 100 ×) with a high numerical aperture (NA) (0.75) could improve the resolution, but also cause expansion of the developed area. For a prolonged exposure time, two electrode patterns merged into a gapless feature. For the 100× magnification, the finest features were obtained during the short exposure time (0.4–0.6 s); the minimum electrode width and gap were 2 and 0.7 μm, respectively. To check the minimum gap, we also fabricated the gap electrodes with various settings (Supplementary Fig. 7). The minimum gap attained with the 100× magnification reached ~ 0.57 μm, which is suitable for device fabrication using small nanosheets with a lateral size of 1–2 μm.

### Possible damage induced by the LED lithography

Processing damage is an important issue for realizing the full potential of 2D nanosheets. Conventional EB lithography often causes inadvertent impurity doping and crystal damage, which deteriorate the electronic properties. In this context, the LED lithography is quite unique. Since this method uses the blue LED component, mild lithography can be achieved, free from the possible beam damage often encountered in the EB lithography.

We now consider chemical damage during the LED lithography. Possible damage comes from the residue of HMDS. In our process, the HMDS is deposited between the substrate and photoresist as a hydrophobic treatment. Since the hydrophilic silanol groups on the substrate would cause the overpenetration of the developer into the interface between the photoresist layer and substrate, the exposed pattern might be ruined by the swelling of the photoresist layer. The (H_3_C)_3_Si group of HMDS would bond with the hydroxy groups at the sample surface to avoid potential damage. Since such (H_3_C)_3_Si group can hardly be removed by the normal lift-off process, the influence of HMDS residue on the nanosheet surface needs to be taken into consideration.

**Figure 4:**
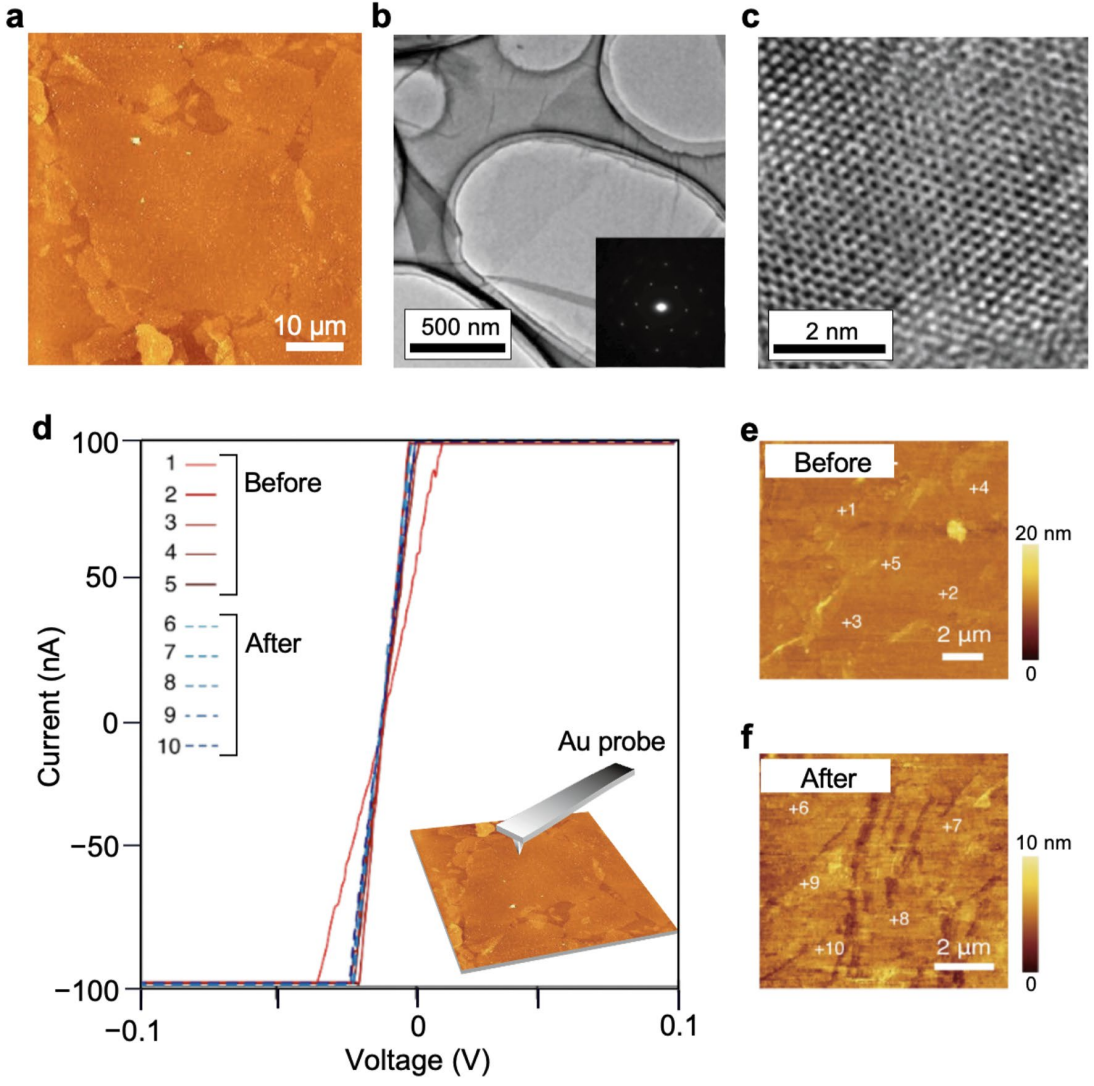
Characterization of possible damages induced by the LED lithography. (**a**) AFM image of a monolayer RuO_2_ nanosheet, (**b**) Low magnification TEM image, (inset) SAED, and (**c**) high-resolution TEM image. (**d–f**) Conductive AFM measurements of monolayer RuO_2_ films before and after the LED lithography. (**d**) I−V curves measured at a series of locations shown in (**e,f**).

To check this conjecture, we investigated the influence of the LED lithography on conducting RuO_2_ nanosheets. RuO_2_ is suitable for this purpose; it exhibits highly conductivity with redox activity^11^, which is sensitive to surface damage and/or contamination. We synthesized RuO_2_ nanosheets by solution-based chemical exfoliation^11^. Characterization by transmission electron microscopy (TEM) and atomic force microscopy (AFM) revealed high-quality single-crystalline monolayers (with a thickness of 1 nm and a lateral size over 10 μm) (Fig. 4a–c). The atomic structure of the RuO_2_ nanosheet was characterized by selected area electron diffraction (SAED) and high-resolution TEM (HRTEM). The SAED and HRTEM image (Fig. 4b,c) showed a planar honeycomb-like atomic structure for monolayer RuO_2_ in similar to the previous reports^12^. We employed conductive atomic force microscopy (c-AFM) to monitor the conducting properties of monolayer nanosheets before and after the LED lithography (Fig. 4d–f). From AFM images (Fig. 4e,f), the nanosheet structure remained without any damage or residue. I − V curves were measured at the series of locations shown in the AFM images (Fig. 4e,f). The conducting property was almost identical at different points before and after the LED lithography; the I − V curves overlapped with the original curve with an error of 1.2%. Based on these results, our method offers soft lithography that does not influence the surface, structure, or metallic contacts of 2D nanosheets.

### LED lithography for 2D nanosheet devices

**Figure 5:**
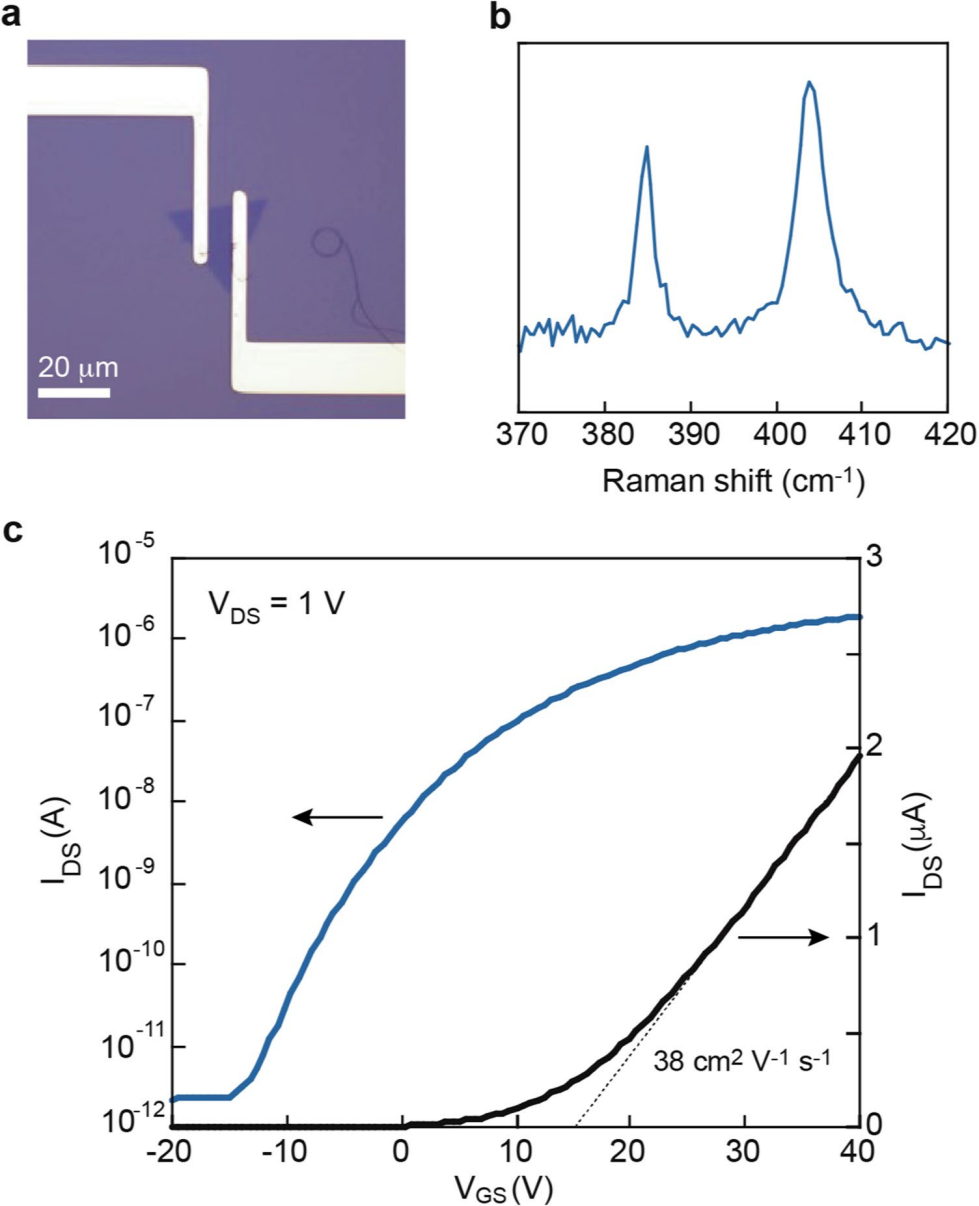
FET devices fabricated by the LED lithography. (**a**) 1 L MoS_2_ FET on a 290 nm-SiO_2_/Si. (**b**) Raman spectrum taken from a monolayer MoS_2_ device. (**c**) Transfer curves measured from a monolayer MoS_2_ device at room temperature.

To investigate the utility of the LED lithography, we performed characterization of nanosheet devices. As a first test, we fabricated a filed effect transistor (FET) device of a monolayer (1L) MoS_2_ nanosheet (Fig. 5a). To check the layer number and possible damage, we performed Raman spectroscopy (Fig. 5b). In 1L MoS_2_, two modes at 385 and 405 cm^−1^ were observed; the peak positions and spectral features are characteristic of 1L MoS_2_ (ref.^13^). Figure 5c shows the FET characteristics of the 1L device. A gate voltage was applied from − 20 to 40 V. This device showed typical n-type behavior with an ON/OFF ratio of ~ 10^6^. From a linear fitting of the transfer curve, the mobility of 1L MoS_2_ was calculated as ~ 38 cm^2^ V^−1^ s^−1^, which was comparable to that of previous reports^4,14^.

**Figure 6:**
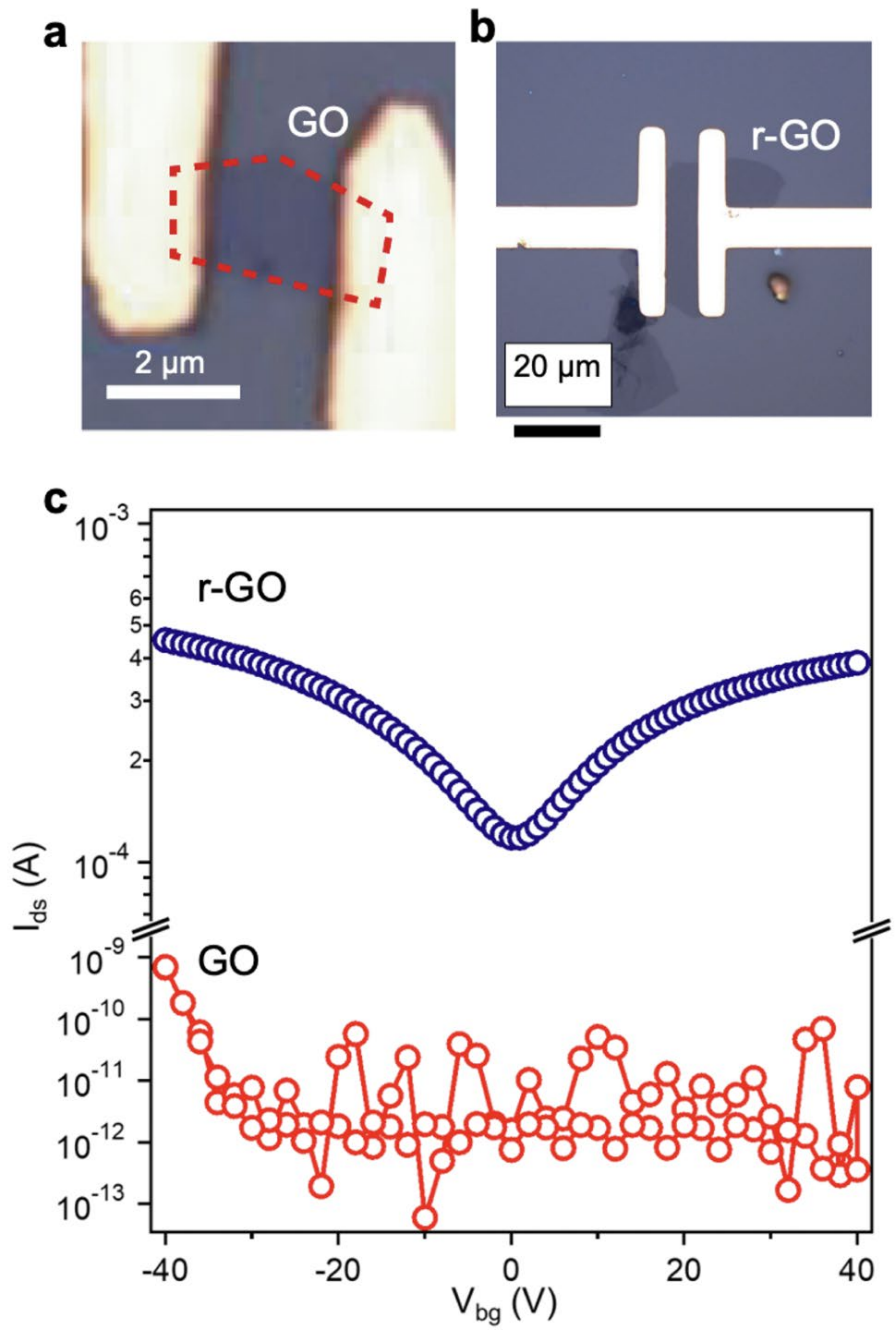
GO devices fabricated by the LED lithography. (**a**) GO FET. (**b**) r-GO FET. (**c**) Transfer curves measured from GO and r-GO FET devices at room temperature.

The advantage of this method is damage-free lithography, which is suitable for device fabrication of weaker materials, such as graphene oxides (GO) and reduced GO (r-GO) (Fig. 6, Supplementary Fig. 8). GO nanosheets were synthesized by a modified Hummers’ method^15,16^. The subsequent thermal reduction at 400 °C in an Ar atmosphere produced r-GO nanosheets. AFM measurements revealed that the nanosheet structure remained even after thermal reduction. The thickness was reduced from 0.9 nm (GO) to 0.5 nm (r-GO), indicating effective reduction (Supplementary Fig. 8a–d). Then, a rectangular area of 1.2 μm^2^ was developed on both samples by the LED lithography. We performed Raman spectroscopy to check the phase stability of the developed areas (Supplementary Fig. 8d). Both GO and r-GO exhibited two modes at ~ 1380 and ~ 1580 cm^−1^, which are characteristic of graphene and graphene-like materials. The D band (~ 1380 cm^−1^) originates from sp^3^ carbon, edges and defects, whereas the G band (~ 1580 cm^−1^) is assigned to the E_2g_ mode of sp^2^ carbon^17,18^. The intensity ratio of two bands (I_D_/I_G_) decreased from 1.2 (GO) to 1.1 (r-GO) with thermal reduction, indicting improved crystallinity. We also note no obvious difference in the Raman spectra of both GO and r-GO even after the LED lithography. This again indicates no distinct damage caused by the LED lithography. Figure 6c shows the FET characteristics of the GO and r-GO devices. A gate voltage was applied from − 40 to 40 V. GO-FET showed low current over the applied range of gate voltage, demonstrating that GO nanosheet was not reduced by LED lithography. GO nanosheet retained a highly insulating nature due to the lack of percolating pathways between sp^2^ carbon clusters. In contrast, the conductivity of r-GO was considerably enhanced due to the partially restoration of the graphene structures. Clearly, r-GO exhibited a high electron mobilities. From a linear fitting of the transfer curve, the mobility was calculated as 7.5 cm^2^ V^−1^ s^−1^. The polarity was switched at approximately 4 V, indicating an ambipolar nature with an ON/OFF ratio of 5.48. These results indicate the applicability of the LED lithography for fabricating nanodevices with reduced nanosheets.

**Figure 7:**
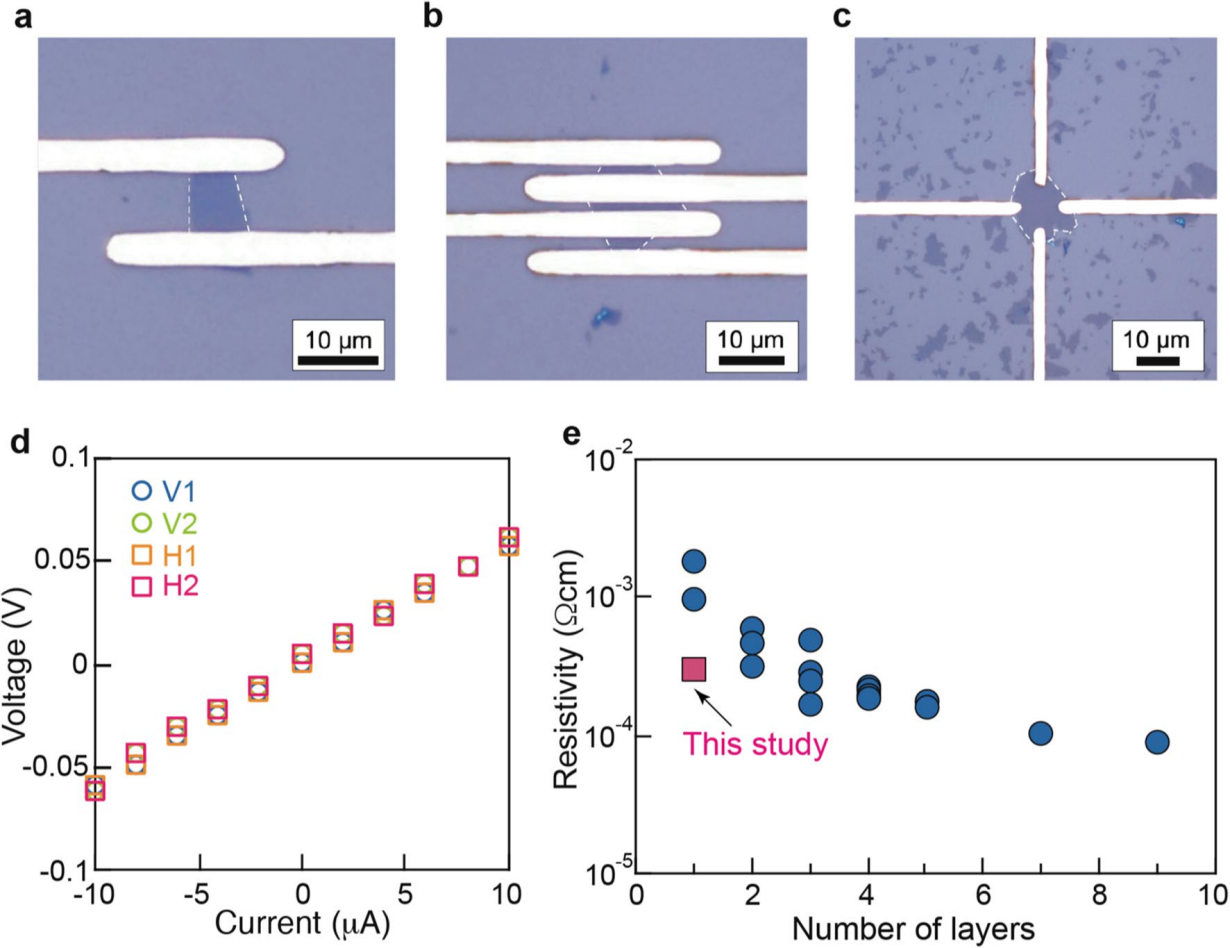
Monolayer RuO_2_ nanosheet devices and their electrical characterization. (**a**) Two-terminal FET device. (**b**) Co-linear four probe device. (**c**) van der Pauw device. (**d**) I−V curves from a monolayer van der Pauw device. (**e**) Resistivity of monolayer devices and thin films.

To further assess the utility of the LED lithography, we investigated the device performance of RuO_2_ nanosheets. Electrical characterization was conducted on three types of monolayer devices, including two-terminal, colinear four-terminal and van der Pauw devices (Fig. 7a–c). From the I − V measurements of the two-terminal device, the RuO_2_ nanosheet exhibited a low resistivity of 3 × 10^−4^ Ωcm. We also utilized the other two devices with four terminals to eliminate the influence of the contact resistance and the probe resistance. In the van der Pauw geometry (Fig. 7d), the devices exhibited a linear I − V response in different configurations, indicating accurate measurements achieved in the ohmic contact between the RuO_2_ nanosheets and metal electrodes. From the van der Pauw devices, similar resistivity value (3 ~ 3.1 × 10^−4^ Ωcm) were observed, indicating that the accurate measurements were achieved in different RuO_2_ devices. We also compared the sheet resistance between 1 L devices and multilayer films^19^ (Fig. 7e). Our devices exhibited higher conductivity than previously reported values^8^. These results again indicate the importance of damage-free lithography for realizing the full potentials of 2D nanosheets.

## Conclusion

We have demonstrated a simple and rapid manufacturing of 2D material devices using the LED lithography. In the LED lithography, the PC-designed pattern can precisely be located and rapidly developed on the photoresist layers by the LED projector so that maskless photolithography can be achieved, free from the process damage often encountered in the conventional lithography. This method is particularly suitable for 2D materials with sensitivity to conventional EB lithography. We applied this LED lithography to device fabrication of selected nanosheets (MoS_2_, GO, r-GO and RuO_2_), and achieved damage-free lithography of various patterned electrodes with feature sizes as small as 1 μm. Our method enables the on-demand device fabrication of 2D nanosheets free from the processing damage, providing a detailed glimpse of the true properties of 2D nanosheets.

Another important aspect is the simplicity of our system. Our LED lithography system is quite simple and inexpensive (~ 7000 US$), simply combining an ordinary optical microscope with a commercially available LED projector. This is the big advantage compared to conventional EB lithography; typical EB lithography systems used in commercial applications are very expensive (> 1 million US$). The LED lithography offers a cost-effective photolithography for device fabrication without any costly instruments, high vacuum, or complex operation.

## Methods

### Preparation of 2D nanosheets

We used some selected nanosheets (MoS_2_, GO, Ti_0.87_O_2_ and RuO_2_) as a model system for the LED lithography.

### MoS_2_ nanosheets

MoS_2_ nanosheets were prepared by either mechanical exfoliation or the chemical vapor deposition (CVD) method. MoS_2_ nanosheets with a few molecular layers could be obtained by repeating the peeling process using Scotch tape. The obtained nanosheets were then be transferred to the target substrate and rubbed with a tool such as an eraser to increase their adhesion to the substrate. After the Scotch tape was removed, MoS_2_ nanosheets were left on the substrate. Monolayer MoS_2_ nanosheets were directly synthesized on a Si substrate by the CVD method^20^.

### Oxide nanosheets

In the GO, Ti_0.87_O_2_ and RuO_2_ cases, colloidal suspensions of 2D nanosheets were used for the LED lithography.

GO nanosheets were synthesized by a modified Hummers’ method^15,16^. Graphite oxide (0.2 g) powder was mixed with deionized water (200 mL). The obtained suspension was sonicated in ice water at 28 kHz for 1 h. Then, the suspension was centrifuged at 6000 rpm, and the upper supernatant was taken as a colloidal suspension.

Ti_0.87_O_2_ and RuO_2_ nanosheets were prepared by a soft-chemical exfoliation method^11,21^. The starting layered compounds (K_0.8_Ti_1.73_Li_0.27_O_4_ and K_0.2_RuO_2.1_) were prepared by a solid-state reaction and converted into their protonated oxides in a HCl solution. The obtained protonated oxides were treated with an aqueous solution of tetrabutylammonium hydroxide (TBAOH), which induced total delamination into nanosheets (Ti_0.87_O_2_ and RuO_2_). The colloidal nanosheet suspensions thus obtained were used for the LED lithography.

We employed dip coating for nanosheet deposition. To remove unexfoliated patches and impurities, the colloidal suspension was centrifuged at 2000 rpm for 15 min, and the supernatant was collected. Then, 100 μL of the suspension was diluted into 50 mL of ultrapure H_2_O as the precursor suspension. Prior to deposition, 90-nm SiO_2_/Si substrates were cleaned by placing them in a mixed solution of CH_3_OH/HCl [1:1 (v/v)] for 30 min, following by 30 min in concentrated H_2_SO_4_. Then, the cleaned substrates were dipped into the precursor suspension and dried at 100 °C for 1 min. In this way, the nanosheets could be dispersed onto the substrate, which facilitated the device fabrication.

### Reduced GO nanosheets

r-GO nanosheets were synthesized by thermal reduction. GO nanosheets were deposited on a 90-nm SiO_2_/Si substrate by drop casting. For thermal reduction, the deposited GO film was heated in a tubular furnace at 400 °C in an Ar atmosphere for 2 h.

### LED lithography

Device fabrication by LED lithography can be mainly divided into three steps: preparation of the photoresist, patterning and metal deposition.

### Preparation of the photoresist layer

The substrates with deposited nanosheets were first prebaked at 90 °C to remove the surface absorbed water. Then, a layer of HMDS was spin-coated at 3000 rpm for 15 s on the substrate as a hydrophobic treatment. Furthermore, AZ1500 photoresist polymer was spin-coated on top of the HMDS layer at 5000 rpm for 60 s. After heating at 90 °C for 3 min, the photoresist layer was prepared.

### Patterning

After preparation of the photoresist layer, the positions of individual nanosheets were located by using optical microscopy. According to the shape and position of nanosheets, the pattern of electrodes could be designed on a PC. Then, the designated pattern was projected, and exposure to safe red light to ensure the focus and the alignment between the designated pattern and the nanosheets. Afterward, the pattern area is exposure to blue light from the projector for 0.1–5 s. After exposure, the substrate was developed in NMD-3 (2.38%) for 1 min and washed in ultrapure water.

### Metal deposition

After the pattern of the electrode was developed, the substrate was coated with 30-nm Au or 5-nm Ti/50-nm Au by using an electron beam deposition system (Sanyu Electron, SVC-700LEB). Then, the substrate was immersed in acetone at 50 °C for 1 h to lift off the extra metal coating. Finally, a nanosheet device was successfully fabricated.

### Characterization

The patterning capability and device structures were characterized by the confocal laser microscopy (Olympus, Lext OLS4000) to investigate the exposure conditions of four different objective lenses (with 10×, 20×, 50× and 100× magnifications). The morphology and structural features of the nanosheets were characterized by AFM (Hitachi, E-Sweep or Oxford Instruments, MFP3D-origin) and TEM (JEOL JEM-2100F). The atomic structures of the RuO_2_ nanosheets were characterized by HRTEM using an accelerating voltage of 80 kV. We employed c-AFM (Hitachi, E-Sweep) to monitor the damage to monolayer nanosheets before and after the LED lithography. The quality of GO nanosheets was verified by Raman spectroscopy (Horiba Jobin Yvon, LabRAM HR-800). The electrical properties of the nanosheet devices were measured using a semiconductor parameter analyzer (Keithley, 4200-SCS) and a probe station (PS-100, Lakeshore).

## Supplementary Information

Supplementary Figures.

## Data Availability

Relevant data supporting the key findings of this study are available within the article and the Supplementary Information file. All data generated during the current study are available from the corresponding authors upon request.

## References

[1] Geim AK, Grigorieva IV (2013). Van der Waals heterostructures. Nature.

[2] Radisavljevic B, Radenovic A, Brivio J, Giacometti V, Kis A (2011). Single-layer MoS_2_ transistors. Nat. Nanotechnol..

[3] Osada M, Sasaki T (2012). Two-Dimensional dielectric nanosheets: Novel nanoelectronics from nanocrystal building blocks. Adv. Mater..

[4] Chhowalla M, Jena D, Zhang H (2016). Two-dimensional semiconductors for transistors. Nat. Rev. Mater..

[5] Akinwande D (2019). Graphene and two-dimensional materials for silicon technology. Nature.

[6] Liu Y, Duan X, Shin H-J, Park S, Huang Y, Duan X (2021). Promises and prospects of two-dimensional transistors. Nature.

[7] Childres I (2010). Effect of electron-beam irradiation on graphene field effect devices. Appl. Phys. Lett..

[8] He YH (2011). Modifying electronic transport properties of graphene by electron beam irradiation. Appl. Phys. Lett..

[9] Garcia A, Jose-Yacaman M, Ponce A (2014). Analysis of electron beam damage of exfoliated MoS_2_ sheets and quantitative HAADF-STEM imaging. Ultramicroscopy.

[10] Technical Datasheet AZ^®^ 1500 Series. *Merck KGaA*. https://www.microchemicals.com/micro/tds_az_1500_series.pdf (2021).

[11] Sugimoto W, Iwata H, Yasunaga Y, Murakami Y, Takasu Y (2003). Preparation of ruthenic acid nanosheets and utilization of its interlayer surface for electrochemical energy storage. Angew. Chem. Int. Ed..

[12] Ko D-S (2018). Understanding the structural, electrical, and optical properties of monolayer h-phase RuO_2_ nanosheets: A combined experimental and computational study. NPG Asia Mater..

[13] Lee C, Yan H, Brus LE, Heinz TF, Hone J, Ryu S (2010). Anomalous lattice vibrations of single- and few-layer MoS_2_. ACS Nano.

[14] Wang QH, Kalantar-Zadeh K, Kis A, Coleman JN, Strano MS (2012). Electronics and optoelectronics of two-dimensional transition metal dichalcogenides. Nat. Nanotechnol..

[15] Hummers WS, Offeman RE (1959). Preparation of graphitic oxide. J. Am. Chem. Soc..

[16] Karim MR (2013). Graphene oxide nanosheet with high proton conductivity. J. Am. Chem. Soc..

[17] Yamaguchi H, Eda G, Mattevi C, Kim H, Chhowalla M (2010). Highly uniform 300 mm wafer-scale deposition of single and multilayered chemically derived graphene thin films. ACS Nano.

[18] De Silva KKH, Viswanath P, Rao VK, Suzuki S, Yoshimura M (2021). New insight into the characterization of graphene oxide and reduced graphene oxide monolayer flakes on Si-based substrates by optical microscopy and raman spectroscopy. J. Phys. Chem. C.

[19] Yoo S (2017). Strong enhancement of electrical conductivity in two-dimensional micrometer-sized RuO_2_ nanosheets for flexible transparent electrodes. Nanoscale.

[20] Li S (2019). Wafer-scale and deterministic patterned growth of monolayer MoS_2_
*via* vapor–liquid–solid method. Nanoscale.

[21] Sasaki T, Watanabe M (1998). Osmotic swelling to exfoliation. Exceptionally high degrees of hydration of a layered titanate. J. Am. Chem. Soc..

