# Supplementary material for: Damage-free LED lithography for atomically thin 2D material devices
Source: Sci Rep. 2023 Feb 14;13:2583. doi: 10.1038/s41598-023-29281-w (PMC9929066; doi:10.1038/s41598-023-29281-w)
Supplement: Supplementary file 1 — Supplementary Figures. [file 41598_2023_29281_MOESM1_ESM.pdf]

Supporting Information

Damage-free LED lithography for atomically-thin 2D material devices

Yue Shi[1], Takaaki Taniguchi[2], Ki-Nam Byun[1], Daiki Kurimoto[1], Eisuke Yamamoto[1], Makoto Kobayashi[1], Kazuhito Tsukagoshi[2] and Minoru Osada[1,2*]

[1] *Institute of Materials and Systems for Sustainability (IMaSS) and Department of Materials Chemistry, Nagoya University, Nagoya 464-8601, Japan*

[2]*International Center for Materials Nanoarchitectonics (WPI-MANA), National Institute for Materials Science (NIMS), Tsukuba 305-0044, Japan*

This PDF file includes:

Fig. S1. Fabrication process of the substrate with address marks.

Fig. S2. Electrode patterns developed by the 10× objective lens with various exposure time.

Fig. S3. Electrode patterns developed by the 20× objective lens with various exposure time.

Fig. S4. Electrode patterns developed by the 50× objective lens with various exposure time.

Fig. S5. Electrode patterns developed by the 100× objective lens with various exposure time.

Fig. S6. Relationship between the electrode interval and exposure time for the electrode patterns fabricated by different objective lenses.

Fig. S7. Au gap electrodes fabricated with various settings (1 ~ 6 μm).

Fig. S8. AFM images and height profiles of GO and r-GO nanosheets. Raman spectra of monolayer devices of GO and r-GO before and after the LED lithography.

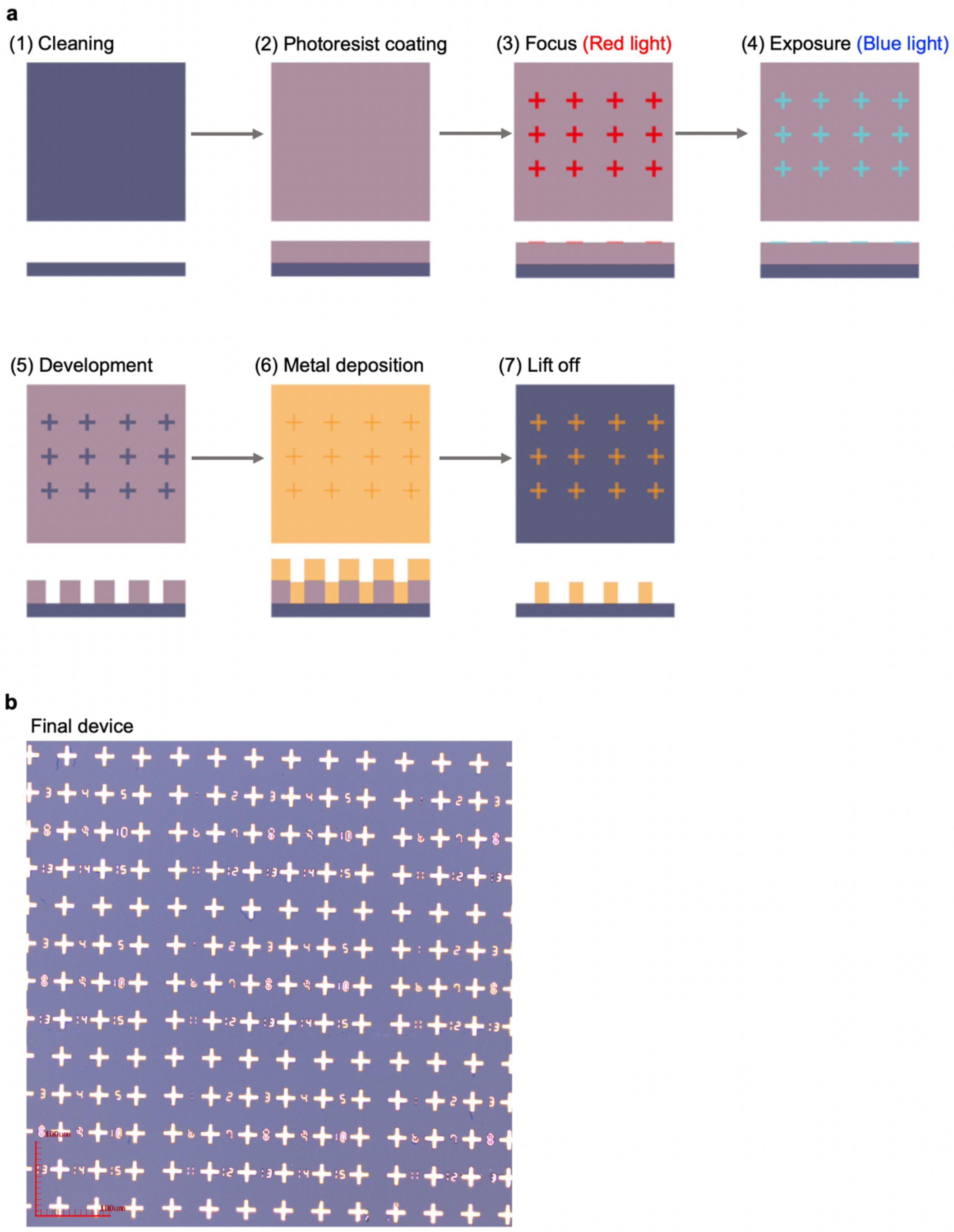

Figure S1. Fabrication process of the substrate with address marks.

(a) Schematic procedure and (b) photograph of Au marks on a 90 nm SiO_2/Si substrate.

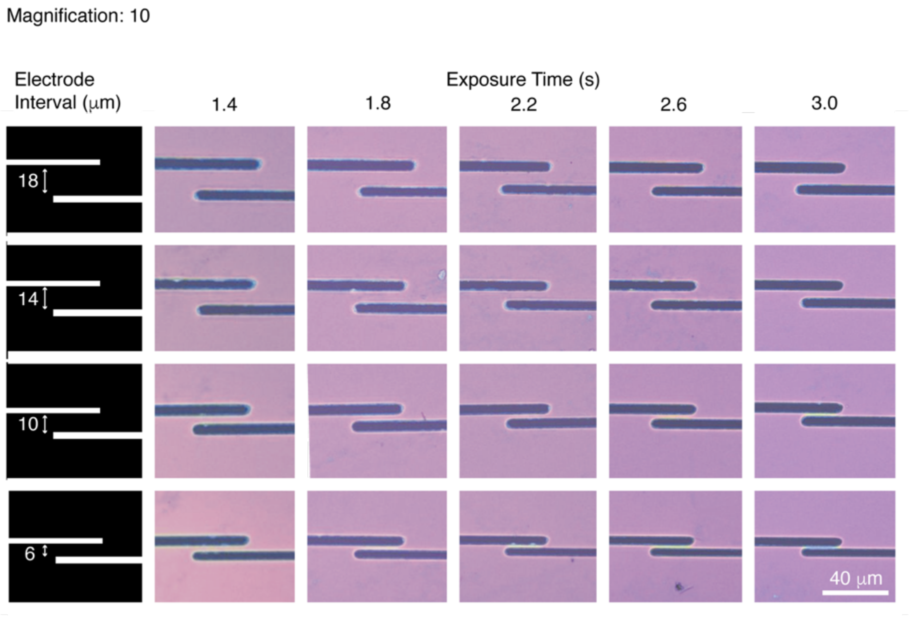

Figure S2. Electrode patterns developed by the 10× objective lens with various exposure time.

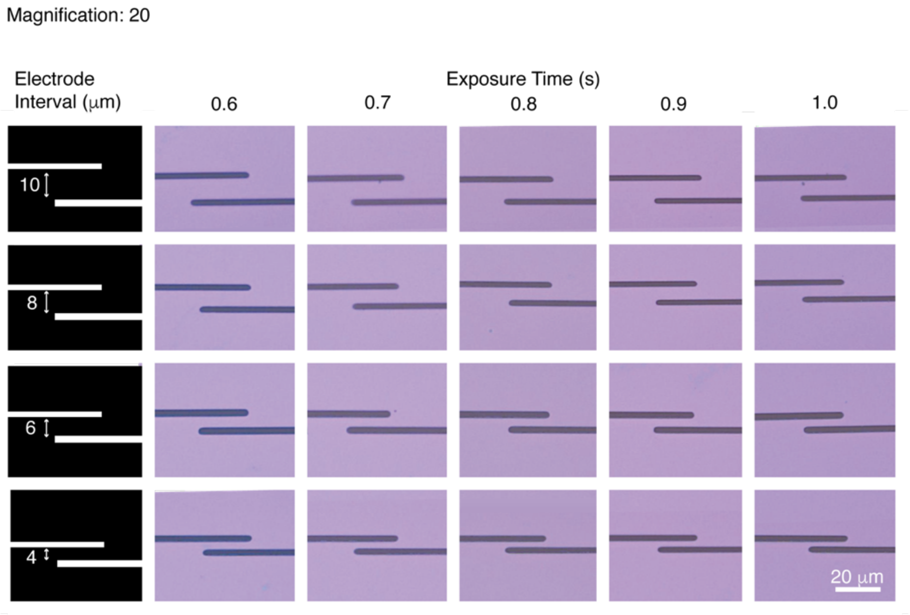

Figure S3. Electrode patterns developed by the 20× objective lens with various exposure time.

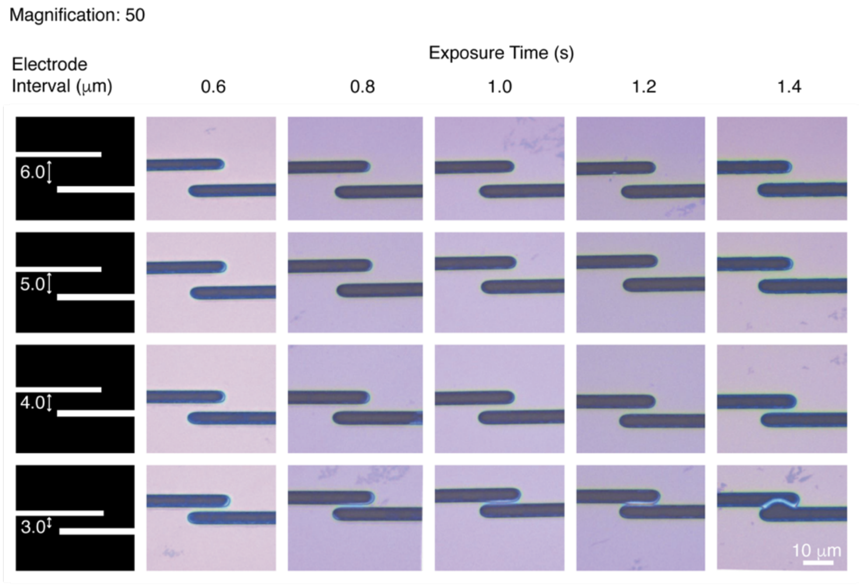

Figure S4. Electrode patterns developed by the 50× objective lens with various exposure time.

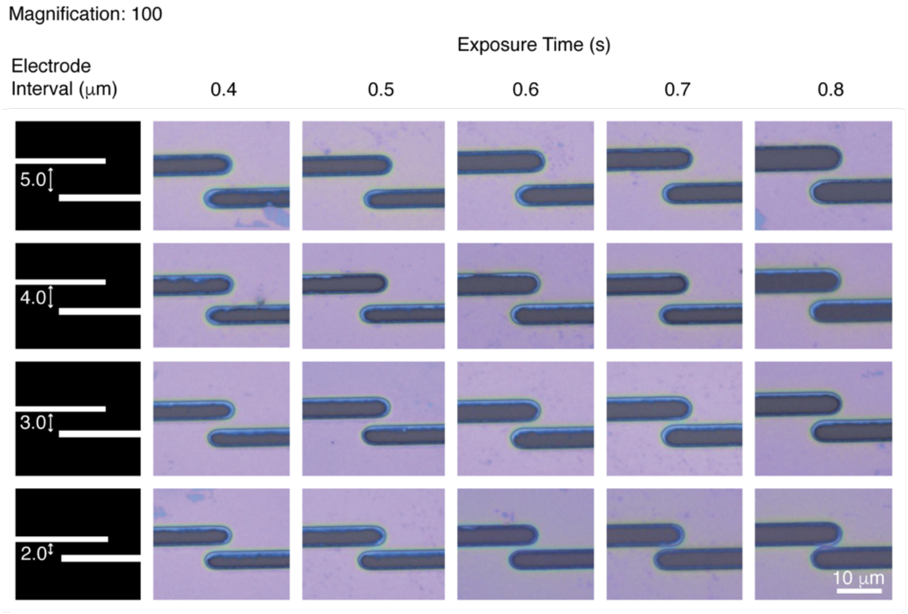

Figure S5. Electrode patterns developed by the 100× objective lens with various exposure time.

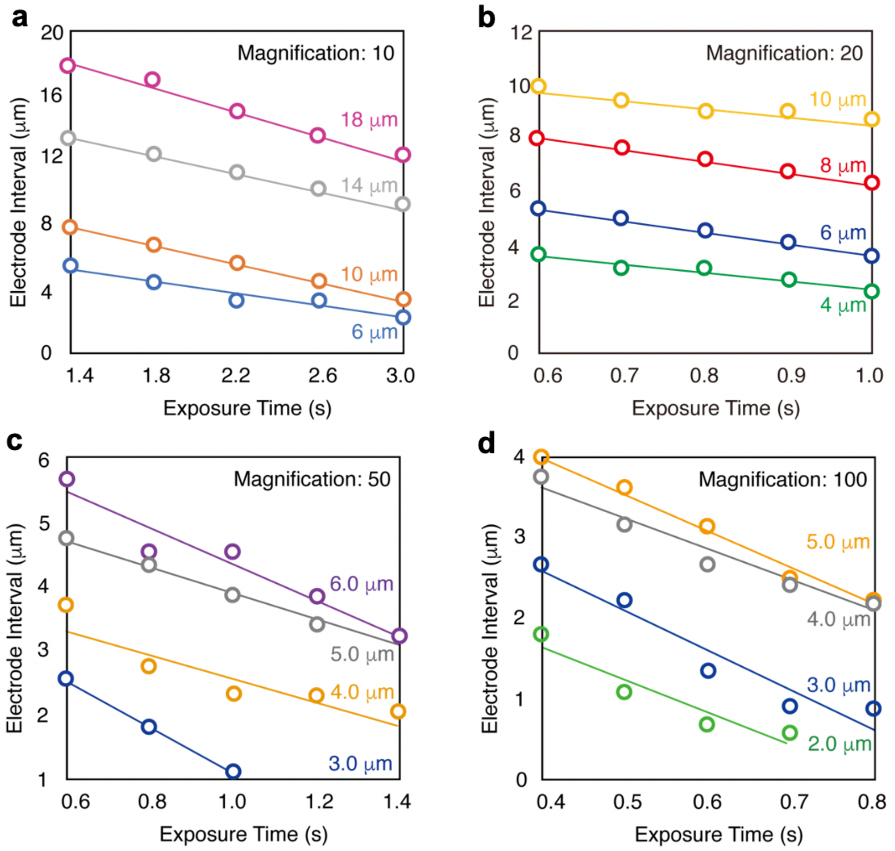

Figure S6. Relationship between the electrode interval and exposure time for the electrode patterns fabricated by different objective lenses: (a) 10×, (b) 20×, (c) 50× and (d) 100×.

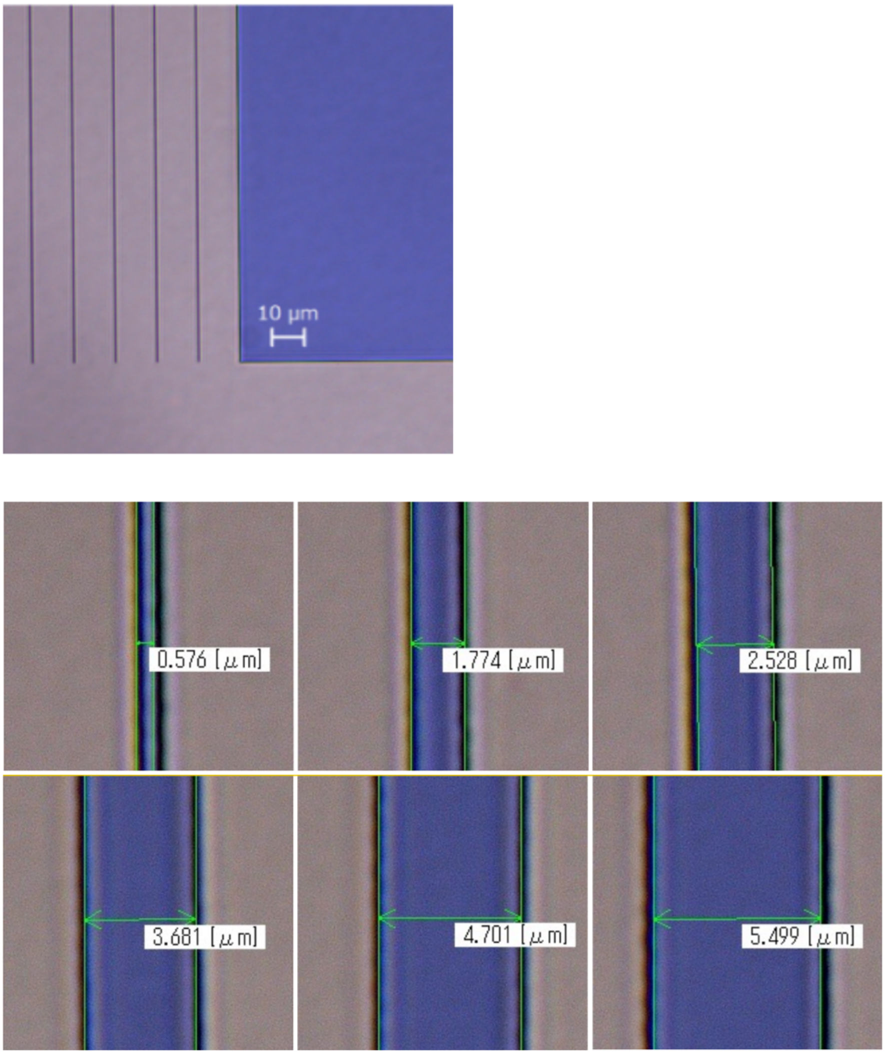

Figure S7. Au gap electrodes fabricated with various settings (1 ~ 6 µm).

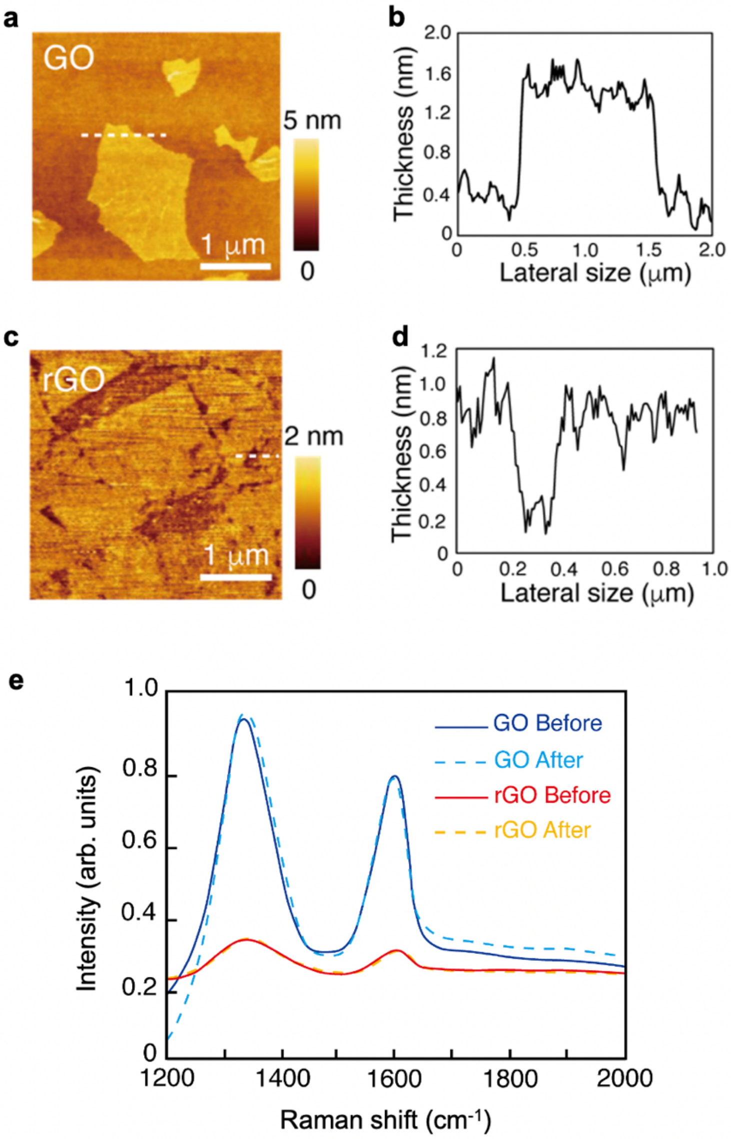

Figure S8. (a, c) AFM images and (b, d) height profiles of GO and r-GO nanosheets. (e) Raman spectra of monolayer devices of GO and r-GO before and after the LED lithography.
